# Supplementary material for: Drug repurposing for aging research using model organisms
Source: Aging Cell. 2017 Jun 16;16(5):1006–15. doi: 10.1111/acel.12626 (PMC5595691; doi:10.1111/acel.12626)
Supplement: Supplementary file 7 — Data S1 Zip‐Archive of all report cards. [file ACEL-16-1006-s007.zip › RC_643.pdf]

643

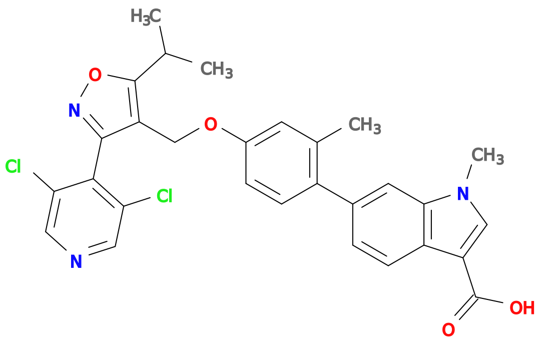

Database identifiers

ChEMBLCompound	CHEMBL466323

Ranking

	Rank	Score
Drosophila	649/697	0.109
C. elegans	NA	NA

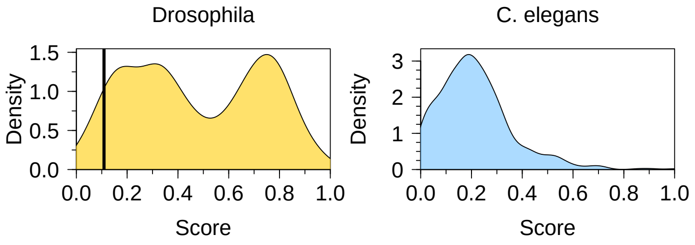

	Ageing implication	Domain conservation	Binding site conservation	Binding affinity	Bioavailability	Lipinski	Promiscuity	Purchasability	Drug approval	**Total**
Drosophila	1.0	0.475	0.507	0.966	(0.9)	-0.1	-0.0	0.0	0.0	0.109
C. elegans	NA	NA	NA	NA	NA	NA	NA	NA	NA	NA

Names

No synonyms found

Roles

ChEBI entry None has no roles

Status

Approved drug (according to ChEMBL)	No
Number of Rule of 5 violations	2
Binding affinity to original target in log units (RF-Score prediction)	8.35
Burns *C. elegans* bioavailability prediction	-1.72

Compound Target Characteristics

Bile acid receptor

Best gene implication in ageing for this target family came from gene P34021 annotated in UniProt release 2014_02. Annotation GO 8340 (determination of adult lifespan) was Inferred from Mutant Phenotype

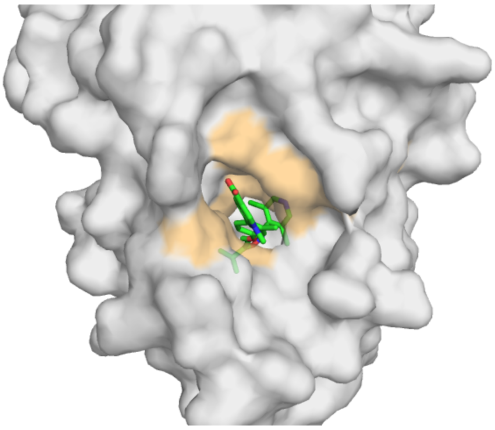

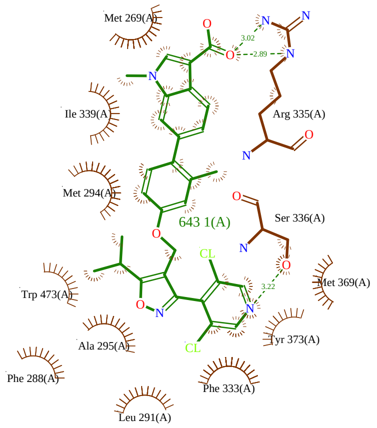

protein	amino acids contacts (binding site)													
PDB:3fxv:chainA:Q96RI1	M	F	L	M	A	M	F	R	S	I	I	M	Y	W
tr:B7Z423:B7Z423_HUMAN	M	F	L	M	A	M	F	R	S	I	I	M	Y	W
tr:F1DAL1:F1DAL1_HUMAN	M	F	L	M	A	M	F	R	S	I	I	M	Y	W
tr:B6ZGS9:B6ZGS9_HUMAN	M	F	L	M	A	M	F	R	S	I	I	M	Y	W
sp:Q96RI1:NR1H4_HUMAN	M	F	L	M	A	M	F	R	S	I	I	M	Y	W
sp:Q62735:NR1H4_RAT	M	F	L	M	A	M	F	R	S	I	I	M	Y	W
tr:D3YTT2:D3YTT2_MOUSE	M	F	L	M	A	M	F	R	S	I	I	M	Y	W
tr:Q3V1T8:Q3V1T8_MOUSE	M	F	L	M	A	M	F	R	S	I	I	M	Y	W
sp:Q60641:NR1H4_MOUSE	M	F	L	M	A	M	F	R	S	I	I	M	Y	W
tr:A4UZ51:A4UZ51_DROME	I	F	I	I	T	M	M	R	M	R	-	L	C	W
sp:P34021:ECR_DROME	I	F	I	I	T	M	M	R	M	R	-	L	C	W
tr:E1JGY2:E1JGY2_DROME	I	F	I	I	T	M	M	R	M	R	-	L	C	W

	whole protein		domain-based		contact-based	
protein	ident	simil	ident	simil	ident	simil
PDB:3fxv:chainA:Q96RI1	0.99	0.99	0.99	0.99	1.0	1.0
tr:B7Z423:B7Z423_HUMAN	0.95	0.95	1.0	1.0	1.0	1.0
tr:F1DAL1:F1DAL1_HUMAN	0.92	0.95	1.0	1.0	1.0	1.0
tr:B6ZGS9:B6ZGS9_HUMAN	0.91	0.94	1.0	1.0	1.0	1.0
sp:Q96RI1:NR1H4_HUMAN	1.0	1.0	1.0	1.0	1.0	1.0
sp:Q62735:NR1H4_RAT	0.86	0.93	0.95	0.99	1.0	1.0
tr:D3YTT2:D3YTT2_MOUSE	0.88	0.93	0.95	0.99	1.0	1.0
tr:Q3V1T8:Q3V1T8_MOUSE	0.92	0.96	0.95	0.99	1.0	1.0
sp:Q60641:NR1H4_MOUSE	0.92	0.97	0.95	0.99	1.0	1.0
tr:A4UZ51:A4UZ51_DROME	0.17	0.37	0.25	0.59	0.29	0.51
sp:P34021:ECR_DROME	0.15	0.34	0.25	0.59	0.29	0.51
tr:E1JGY2:E1JGY2_DROME	0.15	0.34	0.25	0.59	0.29	0.51

EcR (FBgn0000546) associated phenotypes

RU486 conditional, cell death defective, cell shape defective, circadian rhythm defective, conditional, courtship behavior defective, decreased cell death, decreased cell size, developmental rate defective, dominant, feeding behavior defective, heat sensitive, lethal - all die before end of P-stage, lethal - all die before end of first instar larval stage, lethal - all die before end of prepupal stage, lethal - all die before end of pupal stage, locomotor behavior defective, long lived, male fertile, memory defective, mitotic cell cycle defective, neuroanatomy defective, partially, partially lethal - majority die, short lived, size defective, sleep defective, small body, somatic clone, some die during first instar larval stage, some die during pharate adult stage, some die during prepupal stage, some die during pupal

stage, some die during third instar larval stage

(Information from FlyBase)

EcR (UniProt:P34021) annotation
Function: Receptor for ecdysone. Binds to ecdysone response elements (ECRES) following ecdysone-binding, and recruitment of a complex containing the histone methyltransferase trr, leads to activate transcription of target genes. (PubMed:1913820).
Subunit: Heterodimer of USP and ECR. Only the heterodimer is capable of high-affinity binding to ecdysone. Interacts with trr in an ecdysone-dependent manner. (PubMed:14603321, PubMed:8247157).
Subcellular location: Nucleus (PROSITE- ProRule:PRU00407, PubMed:1913820).
Tissue specificity: Isoform B1 predominates over isoform A in larval tissues, imaginal histoblast nests and midgut islands. Isoform A predominates over B1 in imaginal disks, and the larval prothoracic gland. (PubMed:1913820).
Developmental stage: In the salivary glands of mid instar larvae levels increase during puff stage 1 at 86-94 hours of development then remain relatively constant until the premetamorphic pulse of ecdysone in late larvae. Levels diminish dramatically from puff stage 7 onwards. Levels increase in the prepupal period during puff stage 13-14, the level remains stable until stage 21. A decrease in levels at puff stage 7 is also seen in the Malpighian tubules and less dramatically in the fat body and gut. In the wing disk the relatively low level remains unchanged. (PubMed:1913820, PubMed:8223281).

(Information from UniProt)
